# Models for improved diagnosis of left ventricular hypertrophy based on conventional electrocardiographic criteria

**DOI:** 10.1186/s12872-017-0637-8

**Published:** 2017-08-08

**Authors:** Nan Lu, Jin-Xiu Zhu, Pei-Xuan Yang, Xue-Rui Tan

**Affiliations:** 1 Department of Cardiology, The First Affiliated Hospital of Shantou University Medical College, Shantou, Guangdong 515041 China; 2 Health Management Center, The First Affiliated Hospital of Shantou University Medical College, Shantou, Guangdong 515041 China

**Keywords:** Electrocardiogram, Ultrasonic cardiogram, Left ventricular hypertrophy, Diagnostic models

## Abstract

**Background:**

Electrocardiogram (ECG) is commonly used clinically due to convenience, but its accuracy is insufficient for left ventricular hypertrophy (LVH) diagnosis. In this study, we attempted to improve diagnostic accuracy of LVH by establishing models with ECG parameters.

**Methods:**

Eighty hundred and twenty eight patients were recruited in the present study which were divided into groups according to gender, age and body mass index (BMI). The sensitivity, specificity, Youden index, positive predictive value, negative predictive value and accuracy were calculated using ultrasonic cardiogram criteria of LVH as the gold standard. Area under the curve was also calculated to assess the diagnostic accuracy of 22 conventional ECG criteria in different groups. Stepwise discriminant analyses were performed to establish models of ECG for LVH.

**Results:**

The diagnostic accuracy of ECG11 (S V_2_ + R V_5,6_) and ECG12 (S V_1,2_ + R V_5,6_) was significantly higher than the other 20 criteria, while ECG15 (R V_5_/R V_6_) was lowest. The ECG12 sensitivity for males was 52.5%, for <60 years old was 44.2%, and for BMI <25 kg/m^2^ was 46.2%,higher than for females (27.5%), for ≧60 years old (35.7%), and for BMI ≧25 kg/m^2^(27.6%), respectively. The difference between genders was the most obvious. Based on these observations, the following models for males and females were established:

$$ {\displaystyle \begin{array}{c}\hfill {X}_n=0.838\times {SV}_{2,3}+0.742\times {RV}_{5,6}+0.744\times R\kern0.5em aVL\hfill \\ {}\hfill -0.587\times \left( RS\kern0.5em aVF+{RV}_2+{SV}_6\right)\hfill \end{array}} $$

and

$$ Y=0.363\times \mathrm{age}\kern0.5em +\kern0.5em 0.439\times R\  aVL\kern0.5em +\kern0.5em 0.707\times \left(S\kern0.5em {V}_2+R\kern0.5em {V}_{5,6}\right) $$

respectively. The sensitivities of the two new models were 71.4% and 75.8%, significantly higher than the22 conventional ECG criteria.

**Conclusion:**

Two models developed based on gender can be considered for use to investigate the preliminary assessment of the probability of LVH.

**Electronic supplementary material:**

The online version of this article (doi:10.1186/s12872-017-0637-8) contains supplementary material, which is available to authorized users.

## Background

Left ventricular hypertrophy (LVH) is a pathological reaction to underlying cardiovascular disease and is a strong determinant for cardiovascular morbidity and mortality [1–4]. In recent years, research has reported that LVH can be reversed, and associated adverse clinical outcomes can be prevented or delayed with prompt therapy [5]. Therefore, it is important to make early and correct diagnosis for LVH. Currently, LVH is diagnosed either using the standard 12-lead electrocardiogram (ECG) or the more expensive ultrasonic cardiogram (UCG) combined with magnetic resonance imaging, but each protocol has certain limitations [6].

For the diagnosis of LVH, ECG is used much more often than UCG and magnetic resonance imaging because the former is less costly, easier to perform and has high reproducibility [7]. For application of ECG, thirty-six ECG criteria have been recommended [7]. Among them, 22 are based on measurements of QRS voltages that are conventional ECG criteria (Table 1). However, the American Electrocardiogram Committee opined: “Published studies are currently insufficient to indicate whether any of the more recently proposed criteria are clearly superior to the others or are simply redundant.” [7] Furthermore, the various ECG criteria have high specificity, but low sensitivity [8]. Consequently, different modifications in the use of ECG have been proposed [9, 10]. With the various uncertainties, the use of ECG can be compromised. Hence, it is imperative to develop more useful ECG criteria for detection of LVH. The aim of our study was to establish new ECG criteria with higher diagnostic accuracy.

**Table 1:** The 22 conventional ECG criteria of LVH^a^

	Amplitude	First author of study	Year of study publication	Be considered as
Limb lead voltage				
(R I–S I) + (S III–R III)	>16 mm	Lewis	1914	ECG 1
R I + S III	>25 mm	Gubner	1943	ECG 2
R I	>15 mm	Gubner	1943	ECG 3
R aVL	>11 mm	Sokolow	1949	ECG 4
R aVF	>20 mm	Goldberger	1949	ECG 5
Q or S aVR	>19 mm	Schack	1950	ECG 6
R + S in any limb lead	>19 mm	Romhilt	1968	ECG 7
Precordial lead voltage				
S V1	>23 mm	Wilson	1944	ECG 8
S V2	>25 mm	Mazzoleni	1964	ECG 9
S V1 + R V5	>35 mm	Sokolow	1949	ECG 10
S V2 + R V5,6	>45 mm	Romhilt	1969	ECG 11
S V1,2 + R V5,6	>35 mm	Murphy	1984	ECG 12
S V1,2 + R V6	>40 mm	Grant	1957	ECG 13
R + S any precordial lead	>35 mm	Grant	1957	ECG 14
R V5/R V6	>1.0	Holt	1962	ECG 15
R, any precordial lead	>26 mm	McPhie	1958	ECG 16
S V2 + R V4,5	>45 mm	Wolff	1956	ECG 17
R V5	>33 mm	Wilson	1944	ECG 18
R V6	>25 mm	Wilson	1944	ECG 19
Combinations of limb and precordial voltage				
RS aVF + V2 + V6 (>30 years)	>59 mm	Manning	1964	ECG 20
RS aVF + V2 + V6 (<30 years)	>93 mm	Manning	1964	ECG 20
S V3 + R aVL (men)	>28 mm	Casale	1985	ECG 21
S V3 + R aVL (women)	>20 mm	Casale	1985	ECG 21
Total 12-lead voltage	>175 mm	Siegel	1982	ECG 22

Among 36 ECG criteria recommend by the American of electrocardiogram guidelines, 22 based on measurements of QRS voltages (22 conventional ECG criteria) were applied in this study

^a^ *ECG* electrocardiogram, *LVH* Left ventricular hypertrophy; Amplitudes are given in millimeters, where 1 mm = 0.1 mV

## Methods

The study was approved by the First Affiliated Hospital of Shantou University Medical College Institutional Review Board and complied with the local laws (No.071). All subjects provided written informed consent before enrollment in the study.

From October 2013 to April 2014, 828 patients who have performed UCG examination in the First Affiliated Hospital, Shantou University Medical College, were sequentially recruited for participation in the study. Patient demographics (sex, age, height and weight) were collected, and the standard 12-lead ECG and UCG examinations were conducted at the same time. All entrants in our study were Chinese and older than 35 years, as recommended in the 2009 American ECG guidelines [7]. Other exclusion criteria were: having any sign of Q-wave myocardial infarction in the ECG, artificially paced rhythms and pre-excitation syndrome, complete left bundle branch block, complete right bundle branch block, or left anterior fascicular block, as these abnormalities may interfere with ECG determination of LVH [11–13].

For further analyses, the population was divided into six original groups: male and female groups, <60 and ≥60 years old groups (the value of median age was chosen as the cut off for age) [11], and BMI <25 kg/m^2^ and BMI ≥25 kg/m^2^ groups [11, 14]. In addition, the population was organized into male and female groups with further subdivision into <60 years old and ≥60 years old groups. The gender groups were randomly divided into A and B subgroups, in which the characteristics of participants did not differ. Male subgroup A accounted for the 85% of male group and female subgroup A accounted for the 85% of female group. A and B contained 364 and 64 males, and 340 and 60 females, respectively.

### Anthropometrics

Body weights were measured to the nearest 0.1 kg using a medical electronic scale with the subjects wearing indoor clothing. Body height, without shoes, was recorded to the nearest 0.5 cm using a standardized height board. Body mass index (BMI) was calculated as weight/squared height ratio (in kg/m^2^).

### Electrocardiography

A standard 12-lead ECG was recorded during quiet respiration, with subjects in a supine position, at 25 mm/s and 0.1 mV/mm standardization with equipment (NIHON KOHDEN ECG-1350p) whose frequency response characteristics were in accordance with American Heart Association recommendations [7]. The standard 12-lead ECG was recorded by a single technical staff, and assessed by two physicians. The Q-, R-, and S-wave amplitudes of each of the 12 individual ECG leads were extracted directly from the ECG database, using an appropriate software package (Medex, MECG-200 YZB/Jing0092–2011) with visual verification. For the purpose of the present study, we evaluated the 22 conventional ECG criteria based on calculation of QRS voltages mentioned in the recommendations for the diagnosis of LVH (Table 1). These criteria were considered as “ECG1…22” throughout our report.

### Ultrasonic cardiogram

UCG was performed according to standardized procedures as recommended by the American Society of Echocardiography [15]. We used M-mode and two-dimensional echocardiographic measurements from a commercially available instrument (Siemens sc-2000) to calculate the left ventricular mass (LVM). Interventricular septum thickness diastole (IVSTd), left ventricular posterior wall thickness diastole (LVPWTd), and left ventricular internal diameter diastole (LVIDd) were measured from two-dimensionally guided M-mode tracings. LVM was calculated by using the Devereux formula [15, 16]:

$$ LVM=0.8\times \left\{1.04\times \left[{\left( IVSTd\kern0.5em +\kern0.5em LVPWTd\kern0.5em +\kern0.5em LVIDd\right)}^3-{LVIDd}^3\right]\right\}+0.6 $$

To normalize for body surface area (BSA), the LVM index (LVMI) was calculated as LVM/BSA. LVH was defined as LVMI ≥114 g/m^2^ in men, and LVMI ≥99 g/m^2^ in women [14].

### Statistics

Data was analyzed using the SPSS version 16.0. We described continuous variables (age, height, weight, BSA, BMI, IVSTd, LVPWTd, LVIDd, LVM and LVMI) using means ± standard deviations, and categorical variables (gender) using their numbers. Independent-sample T tests, Mann-Whitney U test and Chi-squared test were used to compare the difference of normal continuous variables, non-normal continuous variables and categorical variables, respectively. Sensitivity, specificity, Youden index (Y’s I), positive predictive value (PPV), negative predictive value (NPV) and accuracy were calculated using 2 × 2 tables to assess the diagnostic accuracy of the 22 conventional ECG criteria. Area under the curve (AUC) was also calculated according to receiver operating characteristic (ROC) curve analysis to evaluate the diagnostic accuracy of the 22 conventional ECG criteria. Stepwise discriminant analyses were conducted to establish models of LVH according to male group A and female group A, were used as the training samples, and the diagnostic accuracy of models was tested on male group B and female group B, which were used as the verification samples. We used an entry criterion of *P* < 0.05 and removal criterion of *P* > 0.1.

## Results

### UCG-LVH characteristics of the population

For this study, 828 Chinese patients were successfully recruited. As shown in Table 2,312 (37.7%) patients were found to have a UCG diagnosis of LVH (UCG-LVH) when LVM was indexed to BSA, with 32.9% among males and 42.8% among females. Those with the UCG-LVH positive findings were older, had a lower height, weight and BSA, and greater BMI than those with negative findings. Among these associations (gender, age, height, weight, BSA and BMI), only gender (*P* = 0.004), age (*P* < 0.001) and height (*P* < 0.001) were statistically significant.

**Table 2:** Demographics and UCG parameters of the study population^a^

Group	Whole population	UCG		*P*
		LVH+	LVH-	
N	828	312	516	
Gender (male/female)	428/400	141/171	287/229	0.004
Age (year)	59.79 ± 12.18	62.09 ± 11.55	58.41 ± 12.36	<0.001
Height (cm)	160.26 ± 8.06	158.80 ± 8.15	161.14 ± 7.89	<0.001
Weight (kg)	62.42 ± 11.29	61.89 ± 10.42	62.73 ± 11.78	0.283
BMI (kg/m^2^)	24.24 ± 3.62	24.49 ± 3.33	24.09 ± 3.78	0.115
BSA (m^2^)	1.74 ± 0.17	1.73 ± 0.16	1.75 ± 0.18	0.046
UCG parameters				
IVSd (mm)	11.35 ± 1.89	12.45 ± 2.07	10.68 ± 1.41	<0.001
LVPWd (mm)	10.95 ± 1.56	11.88 ± 1.68	10.38 ± 1.17	<0.001
LVIDd (mm)	44.25 ± 5.62	47.69 ± 5.91	42.16 ± 4.27	<0.001
LVM (g)	177.52 ± 24.06	222.71 ± 52.55	150.20 ± 32.27	<0.001
LVMI (g/m^2^)	101.76 ± 29.00	128.73 ± 26.83	85.45 ± 14.44	<0.001

Demographics and UCG parameters of the UCG-LVH positive and negative groups

^a^Data are shown as means ± SD or absolute numbers. *BMI* body mass index, *BAS* body surface area, *IVSTd* interventricular septum thickness diastole, *LVPWTd* left ventricular posterior wall thickness diastole, *LVIDd* left ventricular internal diameter diastole, *LVM* left ventricular mass, *LVMI* left ventricular mass index, *N* number, *UCG* ultrasound cardiogram

### Diagnostic accuracy of the 22 conventional ECG criteria

#### 2 × 2 tables

**Fig. 1:**
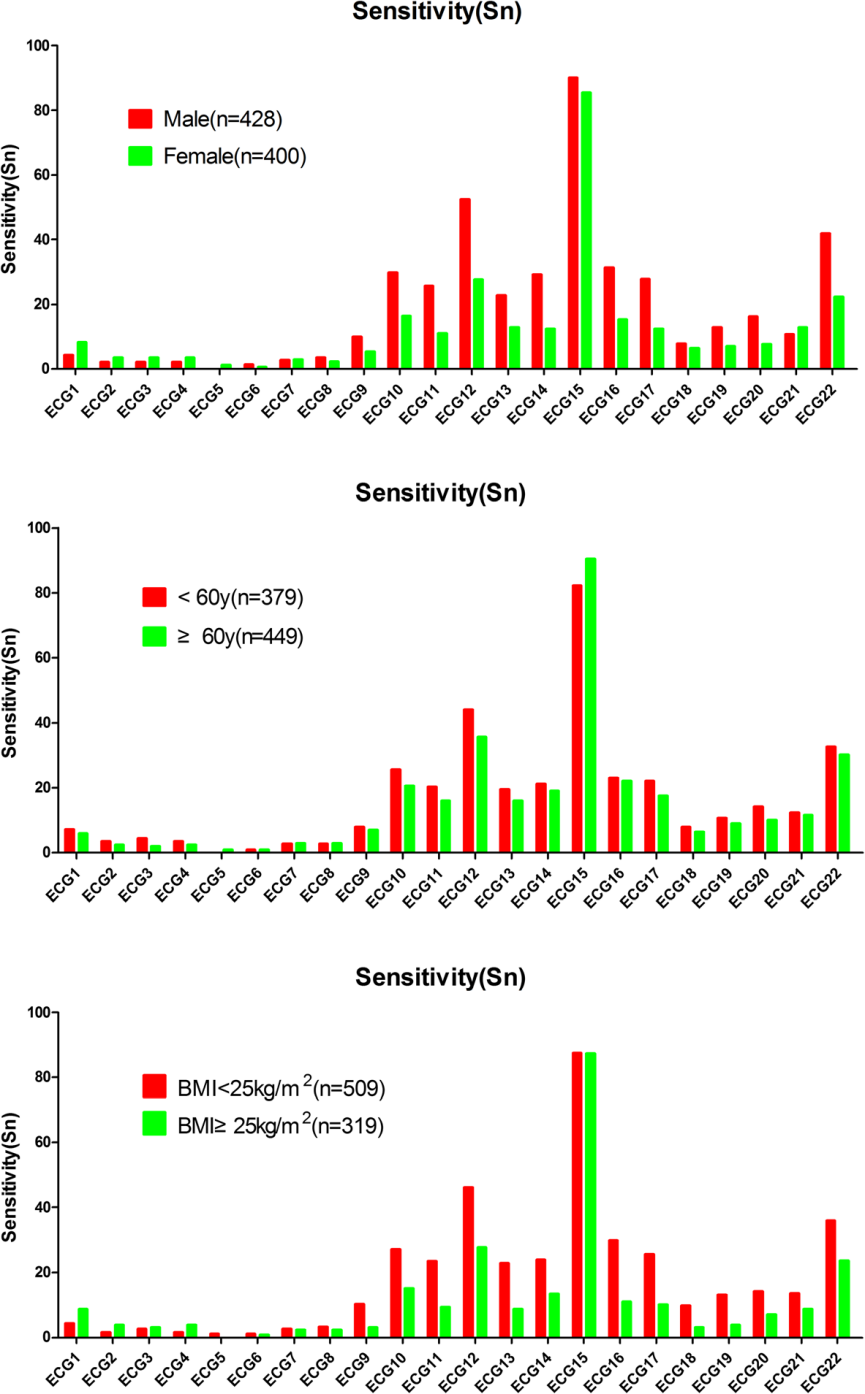
Comparison of sensitivity of the 22 conventional ECG criteria for gender, age, and BMI. The line chart summarizes the comparison of sensitivity of the 22 conventional ECG criteria between males vs. females, age of <60 vs. ≥60 years old, and BMI <25 kg/m^2^ vs. ≥25 kg/m^2^

**Fig. 2:**
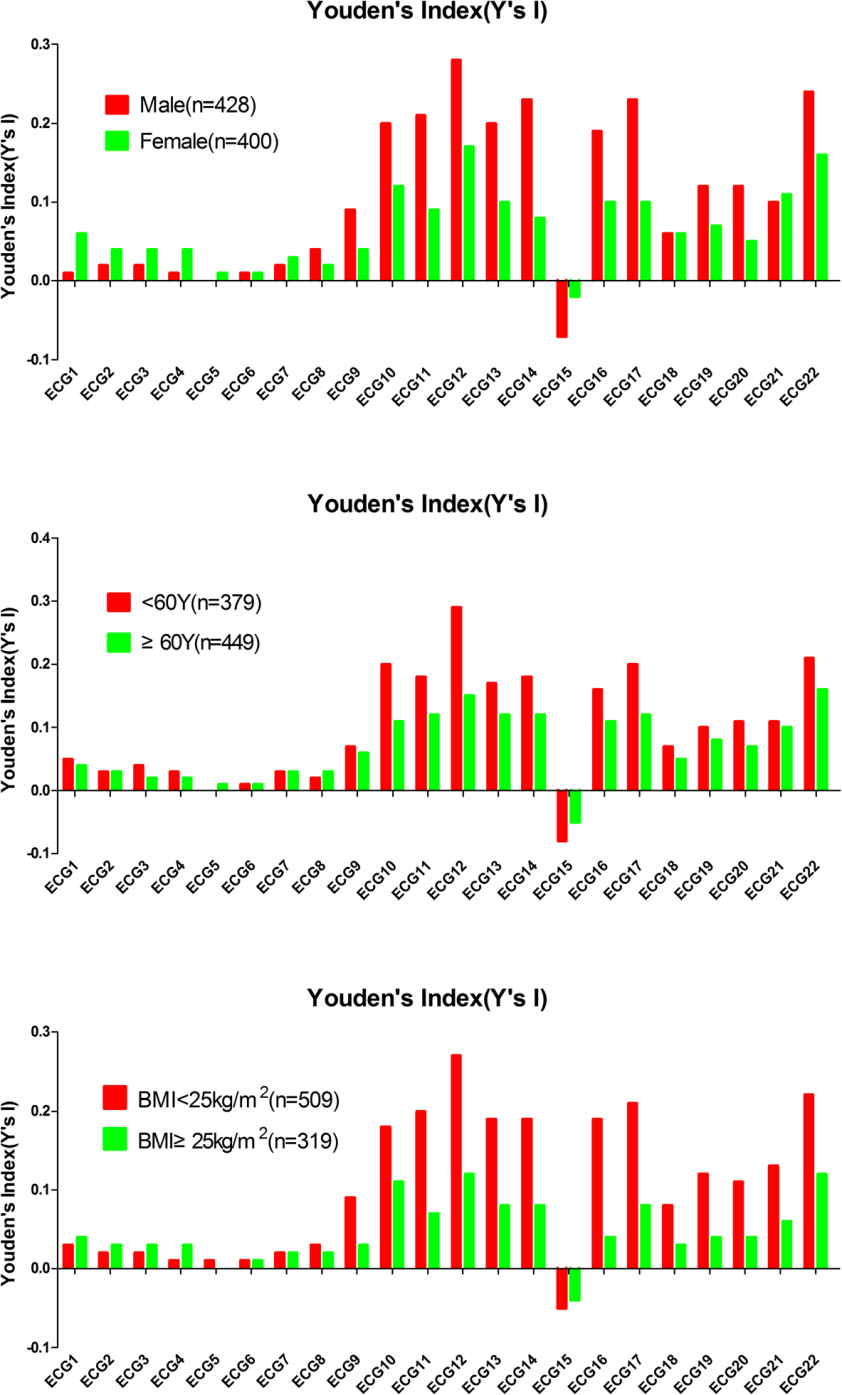
Comparison of Youden indices of the 22 conventional ECG criteria for gender, age, and BMI. The line chart summarizes the comparison of Youden indices of the 22 conventional ECG criteria between males vs. females, ages of <60 vs. ≥60 years old, and a BMI <25 kg/m^2^ vs. ≥25 kg/m^2^ groups

In the six original groups, the values of sensitivity, specificity, Y’s I, PPV, NPV, and accuracy of the 22 conventional ECG criteria for the detection of LVH were evaluated. ECG15 Y’s I less than zero, so it is not recommended used in the diagnosis of LVH and eliminated. Among the 21 remnant conventional ECG criteria, the sensitivity ranged from 0 to 52.5% for gender, age, and BMI. The specificity ranged from 76% to 100%. The Y’s I ranged from 0 to 0.29. The PPV ranged from 42.9% to 100%. The NPV ranged from 55.9% to 78.1%. The accuracy ranged from 56.1% to 74.7%. The sensitivity, NPV, Y’s I of ECG12 was the highest, at52.5%, 78.1%, and0.29 for men, respectively. The findings are highlighted in bar charts (Figs. 1 and 2) and the data are summarized in Additional file 1: Table S1 and Additional file 2: Table S2.

**Fig. 3:**
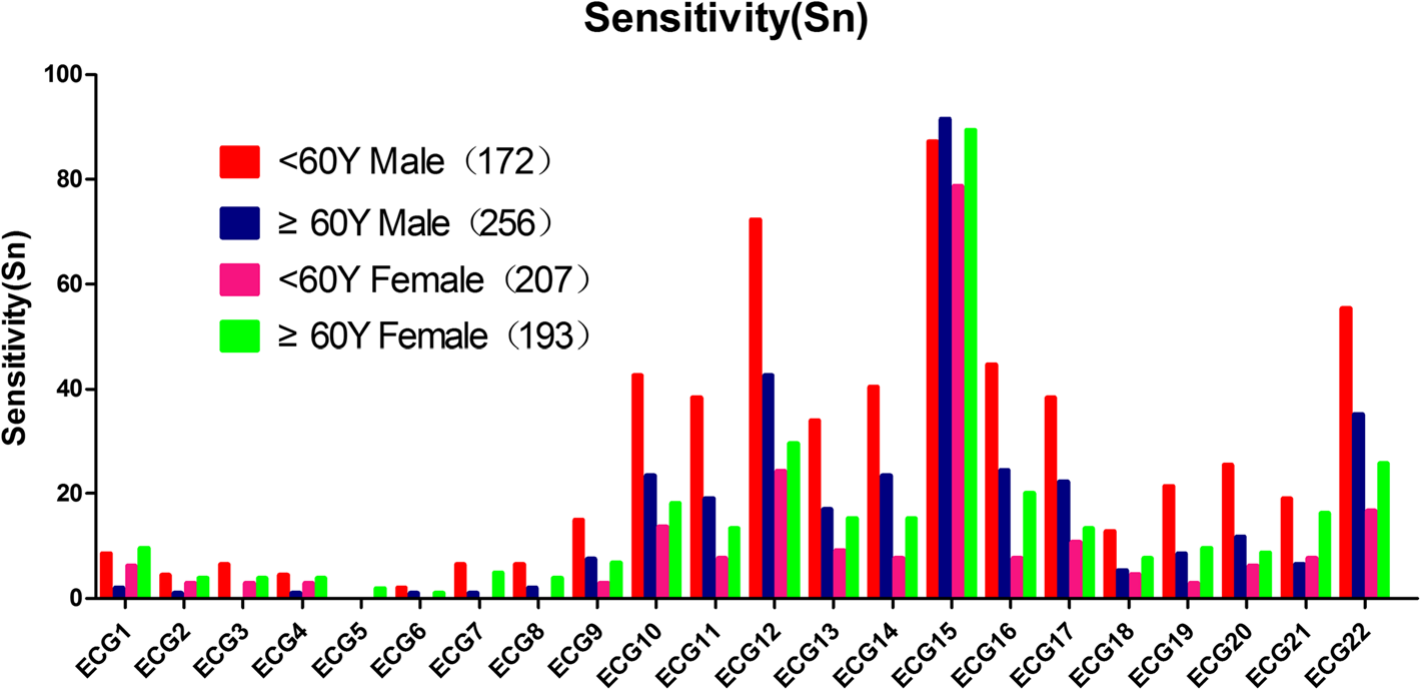
Comparison of sensitivity of the 22 conventional ECG criteria for gender plus age. The line chart summarized the comparison of sensitivity of the 22 conventional ECG criteria in <60 years old male, ≥ 60 years old male groups, < 60 years old female and ≥60 years old female groups

**Fig. 4:**
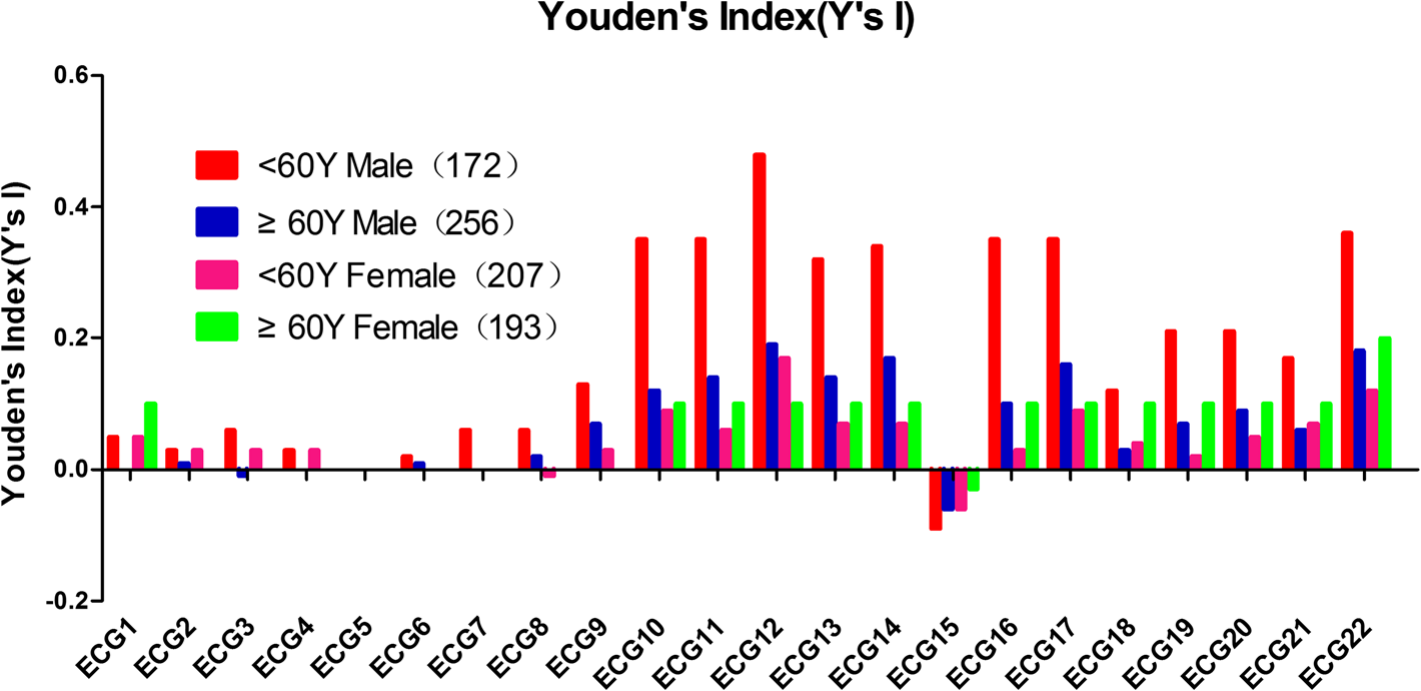
Comparison of the Youden indices of the 22 conventional ECG criteria for gender plus age. The line chart summarizes the comparison of Youden indices of the 22 conventional ECG criteria in males <60 years old and ≥60 years old, and females <60 years old and ≥60 years old

When the overall population was divided into four additional groups according to gender in combination with the two age categories, the values of the six evaluation indicators of the 22 conventional ECG criteria were evaluated. Among the 21 remnent conventional ECG criteria, the sensitivity ranged from 0 to 72.3% for gender plus age. The specificity ranged from 75.2% to 100%. The Y’s I ranged from 0 to 0.48. The PPV ranged from 40.0% to 100%. The NPV ranged from 45.8% to 87.9%. The accuracy ranged from 46.1% to 80.8%. The sensitivity, NPV, Y’s I of ECG12 were the highest, at 72.3%, 87.9%, 0.48, respectively. The diagnosis accuracy of the 22 conventional ECG criteria for LVH has improved significantly using the improved grouping method. Summaries of these evaluations are shown in Figs 3 and 4 and in the two supporting Additional file 3: Table S3 and Additional file 4: Table S4.

In calculating the values of sensitivity, specificity, Y’s I, PPV, NPV, and accuracy in all 10 groups, the diagnostic accuracy of ECG12 was the highest and ECG15 was the lowest among the 22 conventional ECG criteria. Gender, age and BMI influenced the diagnostic accuracy of the 22 conventional ECG criteria, and it will undergo detailed analysis in future studies.

#### ROC curve analysis

**Fig. 5:**
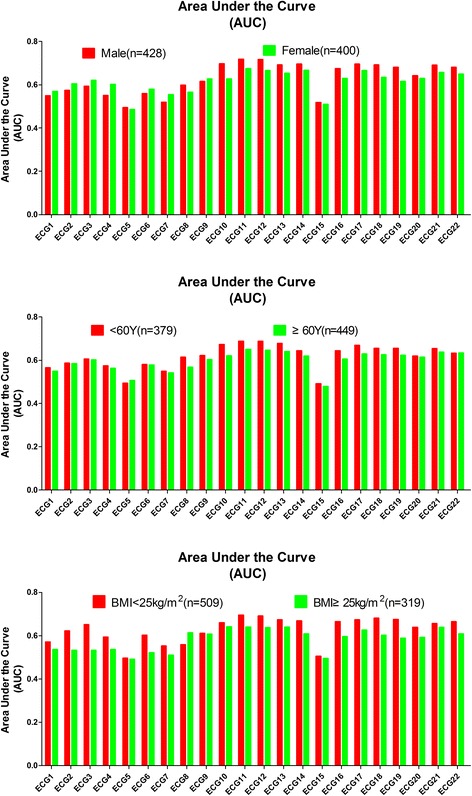
Comparison of the AUCs of the 22 conventional ECG criteria for gender, age and BMI. The line chart summarizes the comparison of AUC of the 22 conventional ECG criteria in males vs. females, ages of <60 years old vs. ≥ 60 years old, and a BMI <25 kg/m^2^ vs. BMI ≥25 kg/m^2^ groups

**Fig. 6:**
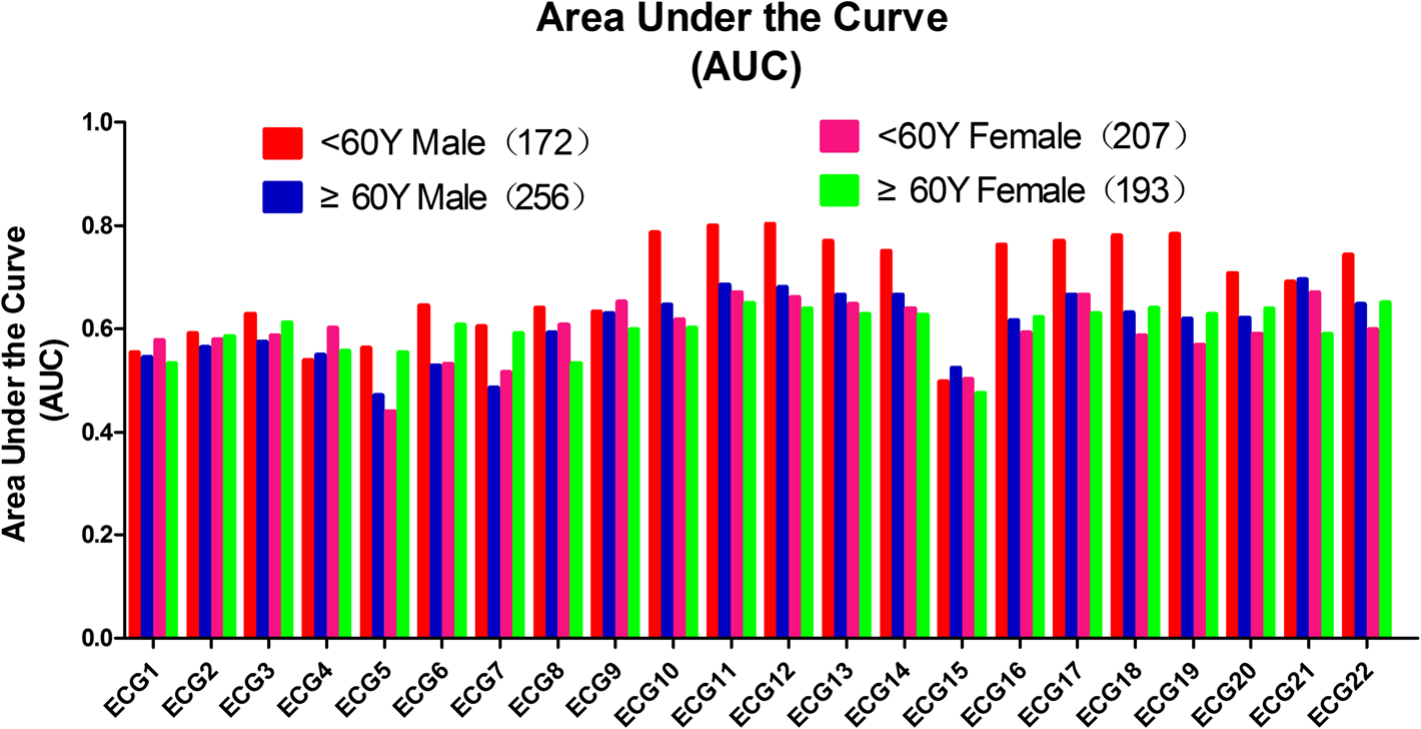
Comparison of AUCs of the 22 conventional ECG criteria for gender plus age. The line chart summarizes the comparison of AUC of the 22 conventional ECG criteria in males <60 years old and ≥60 years old, and females <60 years old and ≥60 years old

The AUC data of the 22 conventional ECG criteria in the 6 original groups and the 4 combined (age-gender) groups are summarized in Figs. 5 and 6, and Additional file 5: Table S5. For the 6 original groups, when the AUC of the 22 conventional ECG criteria were compared within the same groups, the overall performance of ECG11 (S V_2_ + R V_5,6_) was better than that of the other ECG criteria, except in the <60-year-old participants and the BMI ≥25 kg/m^2^ participants, whereas no significant difference was observed between ECG 11 vs. ECG 12, and ECG 11 vs. ECG10.

For the 4 combined groups, when AUC of the 22 conventional ECG criteria were compared on the basis of different sex-age groups, the overall performance of the ECG12, ECG11, ECG21, ECG22 was best for males <60 years old, males ≥60 years old, females <60 years old, and females ≥60 years old,respectively.

The diagnostic accuracy of the 22 conventional ECG criteria was influenced by gender, age and BMI. In ECG12 for example, the sensitivity and AUC of the male group (sensitivity, 52.5%; AUC, 0.717, *P* < 0.001) was higher than that of the female group (sensitivity, 27.5%; AUC, 0.666, *P* < 0.001). The sensitivity and AUC of the <60 years old group (sensitivity, 44.2%; AUC, 0.688, *P* < 0.001) was higher than that of the ≥60 years old group (sensitivity, 35.7%; AUC, 0.646, *P* < 0.001), and the sensitivity and AUC for the BMI <25 kg/m^2^ group (sensitivity, 46.2%; AUC, 0.692, *P* < 0.001) was higher than that for the BMI ≥ 25 kg/m^2^ group (sensitivity, 27.6%; AUC, 0.637, *P* < 0.001). The sensitivity and AUC for <60 years old males (sensitivity, 72.3%; AUC, 0.803, *P* < 0.001) was obviously higher than ≥60 years old males (sensitivity, 42.6%; AUC, 0.681, *P* < 0.001), whereas for females, age did not make a difference in sensitivity, with <60 years old female group (sensitivity, 24.2%; AUC, 0.662, *P* < 0.001) and ≥60 years old female group (sensitivity, 29.5%; AUC, 0.638, *P* < 0.001) showing approximately the same sensitivity.

### Discriminant analysis

ROC curves were made for age, sex, weight, height, BMI, BSA and ECG1 to ECG22 to obtain an impression of their univariate diagnostic performance for LVH diagnosis. The variates that had discriminative power (*P* < 0.05) for LVH were selected to process linear discriminant analysis modeling. From the analyses of males in group A, we developed a model for males:

$$ X=0.613\times ECG9\kern0.5em +\kern0.5em 1.422\times ECG11-0.567\times ECG20+\kern0.5em 0.635\times ECG21 $$

(ECG9 = SV_2_; ECG11 = S V_2_ + R V_5,6_; ECG20 = RS aVF + V_2_ + V_6_; ECG21 = SV_3_ + R aVL). We simplified the model into

$$ {\displaystyle \begin{array}{c}\hfill X=0.681\times {V}_2+\kern0.5em 1.329\times {RV}_{5,6}-0.587\times \left( RS\kern0.5em aVF\kern0.5em +\kern0.5em {V}_2+{V}_6\right)\hfill \\ {}\hfill +0.744\times \left({SV}_3+R\kern0.5em aVL\right)\hfill \end{array}} $$

X < 20.6 predicted negative for LVH, X > 20.6 predicted positive for LVH, and X = 20.6 predicted a critical state. When tested on males in group B, sensitivity was 60.7%, AUC was 0.792, and *P* < 0.001. We further simplified the model, using S V_2,3_ instead of S V_2_ and S V_3_, and used R V_5,6_ instead of R V_5_ and R V_6_, to give

$$ {\displaystyle \begin{array}{c}\hfill {X}_n=0.838\times S\kern0.5em {V}_{2,3}+\kern0.5em 0.742\times R\kern0.5em {V}_{5,6}+\kern0.5em 0.744\times R\kern0.5em aVL\hfill \\ {}\hfill -0.587\times \left( RS\kern0.5em aVF+{RV}_2+{SV}_6\right)\hfill \end{array}} $$

When tested on males in group B, sensitivity was 71.4%, AUC was 0.782, *P* < 0.001. The diagnostic accuracy of the model was significantly higher than both ECG11 (sensitivity, 25.5%; AUC, 0.718, *P* < 0.001) and ECG12 (sensitivity, 52.5%; AUC, 0.717, *P* < 0.001), which had the highest diagnostic accuracy among the 22 conventional ECG criteria in males.

Similarly, we developed a separate model for women based on females in group A:

$$ Y+0.363\times age+0.439\times ECG4+0.707\times ECG11 $$

(ECG4 = R aVL).

We simplified the model as:

$$ Y=0.363\times \mathrm{age}\kern0.5em +\kern0.5em 0.439\kern0.5em R\kern0.5em aVL+0.707\times \left({SV}_2+{RV}_{5,6}\right) $$

Y < 43.0 predicted negative for LVH, Y > 43.0 predicted positive for LVH, and Y = 43.0 predicted a critical state. When tested on females in group B, sensitivity was 75.8%, AUC was 0.792, and *P* < 0.001. The diagnostic accuracy of the model was significantly higher than either ECG11 (sensitivity, 11.1%; AUC, 0.674, *P* < 0.001) or ECG12 (sensitivity, 27.5%; AUC, 0.666, *P* < 0.001), which had the highest diagnostic accuracy among the 22 conventional ECG criteria in females.

## Discussion

Among our patients, 32.9% of males and 42.8% of females were positive for UCG-LVH, with the overall prevalence of UCG-LVH positive cases being 37.7%. Our results are consistent with that in a systematic review which showed ranges from 33% to 65% [8], and 36.6% in males and 53.4% in females in a general population [11]. Among soldiers and pre-soldiers, who should be healthier than the general population, the prevalence of UCG-LVH-positive cases ranged from 1.6% to 11.3% [17].

As reported by other studies, the diagnostic accuracy of the 22 traditional ECG criteria varied from each other in the present study [7]. The sensitivity of ECG12 (SV_1,2_ + RV_5,6_), which had the highest diagnostic accuracy in our groups, is higher than that from other reports showing 1.3% to 21% [14], but close to that of another report showing 43% to 46% sensitivity [18]. Several studies have reported that sensitivity of the conventional ECG criteria would increase as the prevalence and severity of LVH increased [10]. Therefore, the higher sensitivity in our study is likely due to the normal-to-high prevalence of LVH among our patients. Moreover, the population of this study was separated into 10 groups according to gender, age and BMI, which could make our samples more homogeneous, leading to greatly improved sensitivity. The result in the present study was corresponded to previous report [19], with a high sensitivity and a low specificity on ECG15 (RV5:RV6). In the case of LVH, the heart would be rotated which leading an early transition in the precordial leads, and presenting an increased value of RV5:RV6 with a high sensitivity for diagnosis. However, the rotated heart may also exist in many other diseases such as myocardial infarction, the low specificity was showed on ECG15.

In terms of ROC curve analysis: the values of AUC of ECG11 and ECG12 were identified as the highest among the most groups. Nevertheless, in the group of BMI ≥ 25 kg/m^2^ with age ≥ 60 years male and female groups, the values of AUC were shown as the largest on ECG10 (0.641), ECG21 (0.696) and ECG22 (0.651). The values of ECG11 of the above 3 groups were 0.640, 0.685 and 0.649, while on ECG12 were 0.637, 0.681 and 0.638, respectively. The values of AUC of ECG11, ECG12 were very closed to the ones of ECG10, ECG21 and ECG22 in the above three groups. It is indicated that the values of AUC of ECG11 and ECG12 in all groups was relatively high, which is consistent with the results of 2 × 2 tables.

Daniel Pewsner et al. [8] collected 5608 patients from 21 studies to estimate the accuracy of conventional ECG criteria for LVH screening in 2007. The sensibility of S V_1_ + R V_5,6_, R aVL + SV3 and R I + S III was 4–52%, 2–41% and 0–39%, and the specificity of above three criteria was 53–100%, 89–100% and 80–100%, respectively. In addition, the recommendation of the American Electrocardiogram Committee [7] also showed a generally quite low sensitivity and high specificity of the conventional criteria. The results in our study were in accordance with above reports. Low sensitivity of conventional ECG criteria may cause difficulties on clinical application for differentiate diagnosis. Due to the complicated electrophysiological interpretations of LVH [20], controlling some of the important affected factors, such as age, sex and BMI, could improve the diagnosis accuracy of ECG for LVH.

The diagnostic accuracy of the 22 conventional ECG criteria was influenced by gender, age and BMI, as indicated in other reports [10, 21–24]. Under the same criterion, the diagnostic accuracy of the female group was lower than that of the male group. This observation cannot be accounted for by just the heart size between men and women, but also by the thickness of subcutaneous fat layer, various hormones, conductivity of organization and other unknown factors [25].

One significant finding of our study is the demonstration that the diagnostic accuracy of the same conventional ECG criterion is higher in the <60 age group than that of the ≥60 age group. This observation is consistent with two prior reports [11, 25]. This phenomenon may be due to the fact that LVM increases with age, during which muscle tissues are gradually replaced by fibrous tissues, so transmission of electrical activity becomes perturbed. The influence of age may also differ between males and females.

The value of the QRS complex amplitude can be affected by the contact distance between heart and electrode, and the conduction of electrical activity which is also influenced by the thickness of subcutaneous fat layer. The diagnostic accuracy of conventional ECG standard is low in obese people. Similar results were derived from the use of the Sokolow-Lyon standard [26].

The other main finding of this study is that modeling by gender improves performance of ECG for diagnosis of LVH. The above analysis on seven different indicators which suggests diagnostic accuracy of the 22 traditional ECG criteria indicating that gender differences play an important role in ECG criteria. Therefore we modeled based on gender in our study. The test results of Xn and Y show that the diagnostic accuracy of the two models is higher than that of both ECG11 and ECG12, particularly model Y. The model Xn sensitivity for diagnosis LVH was 45.9%, higher than ECG11, and 18.9% higher than ECG12. The model Y sensitivity was 64.7% higher than ECG11, 48.3% higher than ECG12. Y’s I, PPV, NPV, accuracy and AUC of Xn and Y were also higher than that of ECG11 and ECG12.

SV_2_, SV_3_ and R V_5_, R V_6_ respectively represent the value of the cardiac electrical activity in the posterior left and anterior left of horizontal plane [19]. So we regard SV_2_ and SV_3_ as S V_2,3_ (the higher between S V_2_ and S V_3_), and regard RV_5_ and RV_6_as R V_5,6_ (the higher between RV_5_ and RV_6_) to simplify the models. We referred to the report [7] about ECG criteria for diagnosis of LVH from the earliest traditional ECG criteria proposals to more recent innovative ECG criteria. Which indicator of QRS complex amplitude of 12-lead ECG play main responsible for the diagnosis accuracy of ECG criteria, and how do the indicators reflect the differences between males and females, are questions which remain unanswered.

Previous reports do not provide sufficient information on factors that can influence the accuracy of the ECG criteria in the diagnosis of LVH, nor on practical standards after the correction factors have been put forward. In 2012, Sumche Man et al. repeated on 196 cases of outpatients [12]. Univariate analysis and linear discriminant analysis were conducted according to basic information (age, gender, and height) and clinical examination (ECG and vectorcardiogram indicators). The author obtained an ECG diagnosis standard,

$$ D=5.130\times BSA-0.014\times SA-8.74 $$

(SA = vectorcardiogram-derived spatial QRS-T angle), with a diagnostic accuracy of 79%. However, sample size in that study was small, and the vectorcardiogram was not easy to operate. In 2014, Fabio Angeli et al. [10] included 2747 patients with untreated high blood pressure, and recommended correction for the effect of BMI using Cornell voltage standard and multiplication by BMI. Their results showed the prevalence of LVH, sensitivity, specificity and accuracy of the new Cornell standard was 18%, 36.1%, 90.5% and 73.1%, respectively. However, the sensitivity of the new standards is limited. In general, there is no simple solution to correct all variable factors, to improve the diagnostic accuracy, while providing simplicity of calculation. In terms of sensitivity and AUC, we demonstrated that our models performed better than conventional criteria and other new criteria that have been used in clinical practice.

### Limitation

Subjects in this study came from those who needed to have UCG examinations. Therefore, our findings cannot be applied to the general and healthy population. On the other hand, we could not assess all the ECG criteria that were used for the diagnosis of LVH. The larger spectrum of population will be expanded for ECG criteria in our future studies. The cut-off values of LVH vary with ethnicity. For instance, it is lower in Asian populations than those of Europe [27]. Since only Chinese population was investigated in the current study, the cut-off values (114 g/m^2^, 99 g/m^2^) of UCG-LVH were selected according to the PAMELA study of Europe population [14]. Accordingly, it is essential to establish Chinese criteria of UCG-LVH in future studies.

## Conclusions

ECG11 and ECG12 have the highest diagnostic accuracy, while ECG15 has the lowest among the 22 conventional ECG criteria. In addition, gender, age and BMI should be taken into consideration for the ECG criteria of LVH. Two models developed based on gender, have significantly higher diagnostic accuracy than the 22 conventional ECG criteria. Therefore, these models can be considered for use in the preliminary assessment of the probability of LVH.

## Additional files

Additional file 1: Table S1. -Sn, Sp, Y’s I, PPV, NPV and ACC of the 22 conventional ECG criteria for gender, age, and BMI-I. -The Sn, Sp, Y’s I, PPV, NPV and ACC of ECG1 to ECG11 in male and female groups, <60 years old and ≥60 years old groups, and BMI <25 kg/m2 and BMI ≥25 kg/m2 groups. (DOC 83 kb)

Additional file 2: Table S2. Sn, Sp, Y’s I, PPV, NPV and ACC of the 22 conventional ECG criteria for gender, age, and BMI-II. -The Sn, Sp, Y’s I, PPV, NPV and ACC of ECG12 to ECG22 in male and female groups, <60 years old and ≥60 years old groups, and BMI <25 kg/m2 and BMI ≥25 kg/m2 groups. (DOC 85 kb)

Additional file 3: Table S3. Sn, Sp, Y’s I, PPV, NPV and ACC of the 22 conventional ECG criteria for gender plus age-I. The Sn, Sp, Y’s I, PPV, NPV and ACC of ECG1 to ECG11 in <60 years old and ≥60 years old male groups, and <60 years old female and ≥60 years old female groups. (DOC 64 kb)

Additional file 4: Table S4. Sn, Sp, Y’s I, PPV, NPV and ACC of the 22 conventional ECG criteria for gender plus age -II. The Sn, Sp, Y’s I, PPV, NPV and ACC of ECG12 to ECG22 in <60 years old male and ≥60 years old male groups, <60 years old female and ≥60 years old female groups. (DOC 64 kb)

Additional file 5: Table S5. AUC of the 22 conventional ECG criteria for gender, age, and BMI. The AUC of the 22 conventional ECG criteria in male and female groups, <60 years old and ≥60 years old groups, and BMI <25 kg/m2 and BMI ≥ 25 kg/m2 groups; <60 years old male and ≥60 years old male groups, and <60 years old female and ≥60 years old female groups. (DOC 55 kb)

## Abbreviations

AUC: Area under the curve
BMI: Body mass index
ECG: Electrocardiogram
IVSTd: Interventricular septum thickness diastole
LVH: Left ventricular hypertrophy
LVIDd: Left ventricular internal diameter diastole
LVM: Ultrasonic cardiogram
LVPWTd: Left ventricular posterior wall thickness diastole
NPV: Negative predictive value
PPV: Positive predictive value
ROC: Receiver operating characteristic
UCG: Ultrasonic cardiogram
Y’s I: Youdenindex
