# Supplementary material for: Models for improved diagnosis of left ventricular hypertrophy based on conventional electrocardiographic criteria
Source: BMC Cardiovasc Disord. 2017 Aug 8;17:217. doi: 10.1186/s12872-017-0637-8 (PMC5549337; doi:10.1186/s12872-017-0637-8)
Supplement: Supplementary file 1 — -Sn, Sp, Y’s I, PPV, NPV and ACC of the 22 conventional ECG criteria for gender, age, and BMI-I. -The Sn, Sp, Y’s I, PPV, NPV and ACC of ECG1 to ECG11 in male and female groups, <60 years old and ≥60 years old groups, and BMI <25 kg/m2 and BMI ≥25 kg/m2 groups. (DOC 83 kb) [file 12872_2017_637_MOESM1_ESM.doc]

**Additional file 1: Table S1 Sn, Sp, Y's I, PPV, NPV and ACC of the 22 conventional ECG criteria for gender, age, and BMI-Ⅰ.**

|  | ECG  1 | ECG  2 | ECG  3 | ECG  4 | ECG  5 | ECG  6 | ECG  7 | ECG  8 | ECG  9 | ECG  10 | ECG  11 |
| --- | --- | --- | --- | --- | --- | --- | --- | --- | --- | --- | --- |
| Male (n=428) | |  |  |  |  |  |  |  |  |  |  |
| Sn (%) | 4.3 | 2.1 | 2.1 | 2.1 | 0.0 | 1.4 | 2.8 | 3.5 | 9.9 | 29.8 | 25.5 |
| Sp (%) | 97.2 | 99.7 | 99.7 | 99.0 | 100.0 | 100.0 | 99.7 | 100.0 | 99.3 | 90.2 | 95.5 |
| Y's I | 0.01 | 0.02 | 0.02 | 0.01 | 0.00 | 0.01 | 0.02 | 0.04 | 0.09 | 0.20 | 0.21 |
| PPV (%) | 42.9 | 75.0 | 75.0 | 50.0 | - | 100.0 | 80.0 | 100.0 | 87.5 | 60.0 | 73.5 |
| NPV (%) | 67.4 | 67.5 | 67.5 | 67.3 | 67.1 | 67.4 | 67.6 | 67.8 | 69.2 | 72.3 | 72.3 |
| ACC (%) | 66.6 | 67.5 | 67.5 | 67.1 | 67.1 | 67.5 | 67.8 | 68.2 | 69.9 | 70.3 | 72.4 |
| Female (n=400) | |  |  |  |  |  |  |  |  |  |  |
| Sn (%) | 8.2 | 3.5 | 3.5 | 3.5 | 1.2 | 0.6 | 2.9 | 2.3 | 5.3 | 16.4 | 11.1 |
| Sp (%) | 98.3 | 100.0 | 100.0 | 100.0 | 100.0 | 100.0 | 100.0 | 99.6 | 98.7 | 95.2 | 98.3 |
| Y's I | 0.06 | 0.04 | 0.04 | 0.04 | 0.01 | 0.01 | 0.03 | 0.02 | 0.04 | 0.12 | 0.09 |
| PPV (%) | 77.8 | 100.0 | 100.0 | 100.0 | 100.0 | 100.0 | 100.0 | 80.0 | 75.0 | 71.8 | 82.6 |
| NPV (%) | 58.9 | 58.1 | 58.1 | 58.1 | 57.5 | 57.4 | 58.0 | 57.7 | 58.2 | 60.4 | 59.7 |
| ACC (%) | 59.8 | 58.8 | 58.8 | 58.8 | 57.8 | 57.5 | 58.5 | 58.0 | 58.8 | 61.5 | 61.0 |
| ＜60 Y (n=379) | |  |  |  |  |  |  |  |  |  |  |
| Sn (%) | 7.1 | 3.5 | 4.4 | 3.5 | 0.0 | 0.9 | 2.7 | 2.7 | 8.0 | 25.7 | 20.4 |
| Sp (%) | 97.7 | 99.6 | 100.0 | 99.2 | 100.0 | 100.0 | 100.0 | 99.6 | 99.2 | 94.0 | 97.7 |
| Y's I | 0.05 | 0.03 | 0.04 | 0.03 | 0.00 | 0.01 | 0.03 | 0.02 | 0.07 | 0.20 | 0.18 |
| PPV (%) | 57.1 | 80.0 | 100.0 | 66.7 | - | 100.0 | 100.0 | 75.0 | 81.8 | 64.4 | 79.3 |
| NPV (%) | 71.2 | 70.9 | 71.1 | 70.8 | 70.2 | 70.4 | 70.7 | 70.7 | 71.7 | 74.9 | 74.3 |
| ACC (%) | 70.7 | 71.0 | 71.5 | 70.7 | 70.2 | 70.4 | 71.0 | 70.7 | 72.0 | 73.6 | 74.7 |
| ≥60 Y (n=449) | |  |  |  |  |  |  |  |  |  |  |
| Sn (%) | 6.0 | 2.5 | 2.0 | 2.5 | 1.0 | 1.0 | 3.0 | 3.0 | 7.0 | 20.6 | 16.1 |
| Sp (%) | 97.6 | 100.0 | 99.6 | 99.6 | 100.0 | 100.0 | 99.6 | 100.0 | 98.8 | 90.8 | 95.6 |
| Y's I | 0.04 | 0.03 | 0.02 | 0.02 | 0.01 | 0.01 | 0.03 | 0.03 | 0.06 | 0.11 | 0.12 |
| PPV (%) | 66.7 | 100.0 | 80.0 | 83.3 | 100.0 | 100.0 | 85.7 | 100.0 | 82.4 | 64.1 | 74.4 |
| NPV (%) | 56.6 | 56.3 | 56.1 | 56.2 | 55.9 | 55.9 | 56.3 | 56.4 | 57.2 | 59.0 | 58.9 |
| ACC (%) | 57.0 | 56.8 | 56.3 | 56.6 | 56.1 | 56.1 | 56.8 | 57.0 | 58.1 | 59.7 | 60.4 |
| BMI＜25kg/m^2^ (n=509) | | |  |  |  |  |  |  |  |  |  |
| Sn (%) | 4.3 | 1.6 | 2.7 | 1.6 | 1.1 | 1.1 | 2.7 | 3.3 | 10.3 | 27.2 | 23.4 |
| Sp (%) | 99.1 | 100.0 | 99.7 | 99.7 | 100.0 | 100.0 | 99.7 | 99.7 | 98.8 | 90.5 | 96.3 |
| Y's I | 0.03 | 0.02 | 0.02 | 0.01 | 0.01 | 0.01 | 0.02 | 0.03 | 0.09 | 0.18 | 0.20 |
| PPV (%) | 72.7 | 100.0 | 83.3 | 75.0 | 100.0 | 100.0 | 83.3 | 85.7 | 82.6 | 61.7 | 78.2 |
| NPV (%) | 64.7 | 64.2 | 64.4 | 64.2 | 64.1 | 64.1 | 64.4 | 64.5 | 66.0 | 68.7 | 68.9 |
| ACC (%) | 64.8 | 64.4 | 64.6 | 64.2 | 64.2 | 64.2 | 64.6 | 64.8 | 66.8 | 67.6 | 69.9 |
| BMI≥25kg/m^2^(n=319) | | |  |  |  |  |  |  |  |  |  |
| Sn (%) | 8.7 | 3.9 | 3.1 | 3.9 | 0.0 | 0.8 | 2.4 | 2.4 | 3.1 | 15.0 | 9.4 |
| Sp (%) | 95.3 | 99.5 | 100.0 | 99.0 | 100.0 | 100.0 | 100.0 | 100.0 | 99.5 | 95.8 | 97.4 |
| Y's I | 0.04 | 0.03 | 0.03 | 0.03 | 0.00 | 0.01 | 0.02 | 0.02 | 0.03 | 0.11 | 0.07 |
| PPV (%) | 55.0 | 83.3 | 100.0 | 71.4 | - | 100.0 | 100.0 | 100.0 | 80.0 | 70.4 | 70.6 |
| NPV (%) | 61.1 | 60.9 | 60.8 | 60.8 | 60.1 | 60.3 | 60.6 | 60.6 | 60.7 | 62.9 | 61.8 |
| ACC (%) | 60.7 | 61.3 | 61.3 | 61.0 | 60.1 | 60.4 | 61.0 | 61.0 | 61.0 | 63.5 | 62.3 |
| Data are shown as percentages or absolute numbers. ACC=diagnostic accuracy; NPV=negative predictive value; PPV=positive predictive value; Sn=Sensitivity; Sp=Specificity; Y's I=Youden's Index. | | | | | | | | | | | |
