# Supplementary material for: Models for improved diagnosis of left ventricular hypertrophy based on conventional electrocardiographic criteria
Source: BMC Cardiovasc Disord. 2017 Aug 8;17:217. doi: 10.1186/s12872-017-0637-8 (PMC5549337; doi:10.1186/s12872-017-0637-8)
Supplement: Supplementary file 2 — Sn, Sp, Y’s I, PPV, NPV and ACC of the 22 conventional ECG criteria for gender, age, and BMI-II. -The Sn, Sp, Y’s I, PPV, NPV and ACC of ECG12 to ECG22 in male and female groups, <60 years old and ≥60 years old groups, and BMI <25 kg/m2 and BMI ≥25 kg/m2 groups. (DOC 85 kb) [file 12872_2017_637_MOESM2_ESM.doc]

**Additional file 2: Table S2 Sn, Sp, Y's I, PPV, NPV and ACC of the 22 conventional ECG criteria for gender, age, and BMI-Ⅱ.**

|  | ECG 12 | ECG 13 | ECG 14 | ECG 15 | ECG 16 | ECG 17 | ECG 18 | ECG 19 | ECG 20 | ECG 21 | ECG 22 |
| --- | --- | --- | --- | --- | --- | --- | --- | --- | --- | --- | --- |
| Male (n=428) | | | | | | | | | | | |
| Sn (%) | 52.5 | 22.7 | 29.1 | 90.1 | 31.2 | 27.7 | 7.8 | 12.8 | 16.3 | 10.6 | 41.8 |
| Sp (%) | 76 | 97.2 | 93.7 | 3.1 | 87.5 | 95.1 | 98.3 | 99.3 | 96.2 | 99 | 81.9 |
| Y's I | 0.28 | 0.2 | 0.23 | -0.07 | 0.19 | 0.23 | 0.06 | 0.12 | 0.12 | 0.1 | 0.24 |
| PPV (%) | 51.7 | 80 | 69.5 | 31.4 | 55 | 73.6 | 68.8 | 90 | 67.6 | 83.3 | 53.2 |
| NPV (%) | 76.5 | 71.9 | 72.9 | 39.1 | 72.1 | 72.8 | 68.4 | 69.9 | 70.1 | 69.3 | 74.1 |
| ACC (%) | 68.2 | 72.7 | 72.4 | 31.8 | 68.9 | 72.9 | 68.5 | 70.8 | 69.9 | 69.9 | 68.7 |
| Female (n=400) | | | | | | | | | | | |
| Sn (%) | 27.5 | 12.9 | 12.3 | 85.4 | 15.2 | 12.3 | 6.4 | 7 | 7.6 | 12.9 | 22.2 |
| Sp (%) | 90 | 96.9 | 96.1 | 12.7 | 95.2 | 97.4 | 99.1 | 99.6 | 97.8 | 98.3 | 93.4 |
| Y's I | 0.17 | 0.1 | 0.08 | -0.02 | 0.1 | 0.1 | 0.06 | 0.07 | 0.05 | 0.11 | 0.16 |
| PPV (%) | 67.1 | 75.9 | 70 | 42.2 | 70.3 | 77.8 | 84.6 | 92.3 | 72.2 | 84.6 | 71.7 |
| NPV (%) | 62.4 | 59.8 | 59.5 | 53.7 | 60.1 | 59.8 | 58.7 | 58.9 | 58.6 | 60.2 | 61.7 |
| ACC (%) | 63.3 | 61 | 60.3 | 43.8 | 61 | 61 | 59.5 | 60 | 59.3 | 61.8 | 63 |
| ＜60 Y (n=379) | | | | | | | | | | | |
| Sn (%) | 44.2 | 19.5 | 21.2 | 82.3 | 23 | 22.1 | 8 | 10.6 | 14.2 | 12.4 | 32.7 |
| Sp (%) | 84.6 | 97.7 | 96.6 | 10.2 | 93.2 | 97.7 | 99.2 | 99.6 | 97 | 98.5 | 88.3 |
| Y's I | 0.29 | 0.17 | 0.18 | -0.08 | 0.16 | 0.2 | 0.07 | 0.1 | 0.11 | 0.11 | 0.21 |
| PPV (%) | 54.9 | 78.6 | 72.7 | 28 | 59.1 | 80.6 | 81.8 | 92.3 | 66.7 | 77.8 | 54.4 |
| NPV (%) | 78.1 | 74.1 | 74.3 | 57.4 | 74 | 74.7 | 71.7 | 72.4 | 72.7 | 72.6 | 75.6 |
| ACC (%) | 72.6 | 74.4 | 74.1 | 31.7 | 72.3 | 75.2 | 72 | 73.1 | 72.3 | 72.8 | 71.8 |
| ≥60 Y (n=449) | | | | | | | | | | | |
| Sn (%) | 35.7 | 16.1 | 19.1 | 90.5 | 22.1 | 17.6 | 6.5 | 9 | 10.1 | 11.6 | 30.2 |
| Sp (%) | 79.6 | 96.4 | 92.8 | 4.4 | 88.4 | 94.4 | 98 | 99.2 | 96.8 | 98.8 | 85.6 |
| Y's I | 0.15 | 0.12 | 0.12 | -0.05 | 0.11 | 0.12 | 0.05 | 0.08 | 0.07 | 0.1 | 0.16 |
| PPV (%) | 58.2 | 78 | 67.9 | 43 | 60.3 | 71.4 | 72.2 | 90 | 71.4 | 88.5 | 62.5 |
| NPV (%) | 60.9 | 59.1 | 59 | 36.7 | 58.8 | 59 | 56.8 | 57.8 | 57.5 | 58.4 | 60.6 |
| ACC (%) | 60.1 | 60.8 | 60.1 | 42.5 | 59 | 60.4 | 57.5 | 59.2 | 58.4 | 60.1 | 61 |
| BMI＜25kg/m^2^ (n=509) | | | | | | | | | | | |
| Sn (%) | 46.2 | 22.8 | 23.9 | 87.5 | 29.9 | 25.5 | 9.8 | 13 | 14.1 | 13.6 | 35.9 |
| Sp (%) | 80.9 | 96 | 95.1 | 7.1 | 89.5 | 95.4 | 98.2 | 99.1 | 96.9 | 99.4 | 86.5 |
| Y's I | 0.27 | 0.19 | 0.19 | -0.05 | 0.19 | 0.21 | 0.08 | 0.12 | 0.11 | 0.13 | 0.22 |
| PPV (%) | 57.8 | 76.4 | 73.3 | 34.8 | 61.8 | 75.8 | 75 | 88.9 | 72.2 | 92.6 | 60 |
| NPV (%) | 72.7 | 68.7 | 68.8 | 50 | 69.3 | 69.4 | 65.8 | 66.8 | 66.6 | 67 | 70.4 |
| ACC (%) | 68.4 | 69.5 | 69.4 | 36.1 | 68 | 70.1 | 66.2 | 68 | 67 | 68.4 | 68.2 |
| BMI≥25kg/m^2^ (n=319) | | | | | | | | | | | |
| Sn (%) | 27.6 | 8.7 | 13.4 | 87.4 | 11 | 10.2 | 3.1 | 3.9 | 7.1 | 8.7 | 23.6 |
| Sp (%) | 84.8 | 99 | 94.2 | 8.4 | 93.2 | 97.4 | 99.5 | 100 | 96.9 | 97.4 | 88.5 |
| Y's I | 0.12 | 0.08 | 0.08 | -0.04 | 0.04 | 0.08 | 0.03 | 0.04 | 0.04 | 0.06 | 0.12 |
| PPV (%) | 54.7 | 84.6 | 60.7 | 38.8 | 51.9 | 72.2 | 80 | 100 | 60 | 68.8 | 57.7 |
| NPV (%) | 63.8 | 62 | 62.1 | 50 | 61.2 | 62 | 60.7 | 61 | 61.1 | 61.6 | 63.5 |
| ACC (%) | 61.9 | 62.9 | 61.9 | 39.9 | 60.4 | 62.6 | 61 | 61.6 | 61 | 61.9 | 62.6 |
| Data are shown as percentages or absolute numbers. ACC=diagnostic accuracy; NPV=negative predictive value; PPV=positive predictive value; Sn=Sensitivity; Sp=Specificity; Y's I=Youden's Index. | | | | | | | | | | | |
