# Supplementary material for: Models for improved diagnosis of left ventricular hypertrophy based on conventional electrocardiographic criteria
Source: BMC Cardiovasc Disord. 2017 Aug 8;17:217. doi: 10.1186/s12872-017-0637-8 (PMC5549337; doi:10.1186/s12872-017-0637-8)
Supplement: Supplementary file 3 — Sn, Sp, Y’s I, PPV, NPV and ACC of the 22 conventional ECG criteria for gender plus age-I. The Sn, Sp, Y’s I, PPV, NPV and ACC of ECG1 to ECG11 in <60 years old and ≥60 years old male groups, and <60 years old female and ≥60 years old female groups. (DOC 64 kb) [file 12872_2017_637_MOESM3_ESM.doc]

**Additional file 3: Table S3 Sn, Sp, Y's I, PPV, NPV and ACC of the 22 conventional ECG criteria for gender plus age-Ⅰ.**

|  |  | ECG  1 | ECG  2 | ECG  3 | ECG  4 | ECG  5 | ECG  6 | ECG  7 | ECG  8 | ECG  9 | ECG  10 | ECG  11 |
| --- | --- | --- | --- | --- | --- | --- | --- | --- | --- | --- | --- | --- |
| Male | | | | | | | | | | | | |
| ＜60 Y | Sn (%) | 8.5 | 4.3 | 6.4 | 4.3 | 0.0 | 2.1 | 6.4 | 6.4 | 14.9 | 42.6 | 38.3 |
| n=172 | Sp (%) | 96.0 | 99.2 | 100.0 | 98.4 | 100.0 | 100.0 | 100.0 | 100.0 | 98.4 | 92.0 | 96.8 |
|  | Y's I | 0.05 | 0.03 | 0.06 | 0.03 | 0.00 | 0.02 | 0.06 | 0.06 | 0.13 | 0.35 | 0.35 |
|  | PPV (%) | 44.4 | 66.7 | 100.0 | 50.0 | - | 100.0 | 100.0 | 100.0 | 77.8 | 66.7 | 81.8 |
|  | NPV (%) | 73.6 | 73.4 | 74.0 | 73.2 | 72.7 | 73.1 | 74.0 | 74.0 | 75.5 | 81.0 | 80.7 |
|  | ACC (%) | 72.1 | 73.3 | 74.4 | 72.7 | 72.7 | 73.3 | 74.4 | 74.4 | 75.6 | 78.5 | 80.8 |
| ≥60 Y | Sn (%) | 2.1 | 1.1 | 0.0 | 1.1 | 0.0 | 1.1 | 1.1 | 2.1 | 7.4 | 23.4 | 19.1 |
| n=256 | Sp (%) | 98.1 | 100.0 | 99.4 | 99.4 | 100.0 | 100.0 | 99.4 | 100.0 | 100.0 | 88.9 | 94.4 |
|  | Y's I | 0.00 | 0.01 | -0.01 | 0.00 | 0.00 | 0.01 | 0.00 | 0.02 | 0.07 | 0.12 | 0.14 |
|  | PPV (%) | 40.0 | 100.0 | 0.0 | 50.0 | - | 100.0 | 50.0 | 100.0 | 100.0 | 55.0 | 66.7 |
|  | NPV (%) | 63.3 | 63.5 | 63.1 | 63.4 | 63.3 | 63.5 | 63.4 | 63.8 | 65.1 | 66.7 | 66.8 |
|  | ACC (%) | 62.9 | 63.7 | 62.9 | 63.3 | 63.3 | 63.7 | 63.3 | 64.1 | 66.0 | 64.8 | 66.8 |
| Female | | | | | | | | | | | | |
| ＜60 Y | Sn (%) | 6.1 | 3.0 | 3.0 | 3.0 | 0.0 | 0.0 | 0.0 | 0.0 | 3.0 | 13.6 | 7.6 |
| n=207 | Sp (%) | 99.3 | 100.0 | 100.0 | 100.0 | 100.0 | 100.0 | 100.0 | 99.3 | 100.0 | 95.7 | 98.6 |
|  | Y's I | 0.05 | 0.03 | 0.03 | 0.03 | 0.00 | 0.00 | 0.00 | -0.01 | 0.03 | 0.09 | 0.06 |
|  | PPV (%) | 80.0 | 100.0 | 100.0 | 100.0 | - | - | - | 0.0 | 100.0 | 60.0 | 71.4 |
|  | NPV (%) | 69.3 | 68.8 | 68.8 | 68.8 | 68.1 | 68.1 | 68.1 | 68.0 | 68.8 | 70.3 | 69.5 |
|  | ACC (%) | 69.6 | 69.1 | 69.1 | 69.1 | 68.1 | 68.1 | 68.1 | 67.6 | 69.1 | 69.6 | 69.6 |
| ≥60 Y | Sn (%) | 9.5 | 3.8 | 3.8 | 3.8 | 1.9 | 1.0 | 4.8 | 3.8 | 6.7 | 18.1 | 13.3 |
| n=193 | Sp (%) | 96.6 | 100.0 | 100.0 | 100.0 | 100.0 | 100.0 | 100.0 | 100.0 | 96.6 | 94.3 | 97.7 |
|  | Y's I | 0.06 | 0.04 | 0.04 | 0.04 | 0.02 | 0.01 | 0.05 | 0.04 | 0.03 | 0.12 | 0.11 |
|  | PPV (%) | 76.9 | 100.0 | 100.0 | 100.0 | 100.0 | 100.0 | 100.0 | 100.0 | 70.0 | 79.2 | 87.5 |
|  | NPV (%) | 47.2 | 46.6 | 46.6 | 46.6 | 46.1 | 45.8 | 46.8 | 46.6 | 46.4 | 49.1 | 48.6 |
|  | ACC (%) | 49.2 | 47.7 | 47.7 | 47.7 | 46.6 | 46.1 | 48.2 | 47.7 | 47.7 | 52.8 | 51.8 |
| Data are shown as percentages or absolute numbers. ACC=diagnostic accuracy; NPV=negative predictive value; PPV=positive predictive value; Sn=Sensitivity; Sp=Specificity; Y's I=Youden's Index. | | | | | | | | | | | | |
|  | | | | | | | | | | | | |
