# Supplementary material for: Models for improved diagnosis of left ventricular hypertrophy based on conventional electrocardiographic criteria
Source: BMC Cardiovasc Disord. 2017 Aug 8;17:217. doi: 10.1186/s12872-017-0637-8 (PMC5549337; doi:10.1186/s12872-017-0637-8)
Supplement: Supplementary file 4 — Sn, Sp, Y’s I, PPV, NPV and ACC of the 22 conventional ECG criteria for gender plus age -II. The Sn, Sp, Y’s I, PPV, NPV and ACC of ECG12 to ECG22 in <60 years old male and ≥60 years old male groups, <60 years old female and ≥60 years old female groups. (DOC 64 kb) [file 12872_2017_637_MOESM4_ESM.doc]

**Additional file 4: Table S4 Sn, Sp, Y's I, PPV, NPV and ACC of the 22 conventional ECG criteria for gender plus age -Ⅱ.**

|  |  | ECG  12 | ECG  13 | ECG  14 | ECG  15 | ECG  16 | ECG  17 | ECG  18 | ECG  19 | ECG  20 | ECG  21 | ECG  22 |
| --- | --- | --- | --- | --- | --- | --- | --- | --- | --- | --- | --- | --- |
| Male | | | | | | | | | | | | |
| ＜60 Y | Sn (%) | 72.3 | 34.0 | 40.4 | 87.2 | 44.7 | 38.3 | 12.8 | 21.3 | 25.5 | 19.1 | 55.3 |
| n=172 | Sp (%) | 75.2 | 97.6 | 93.6 | 4.0 | 90.4 | 96.8 | 99.2 | 100.0 | 95.2 | 97.6 | 80.8 |
|  | Y's I | 0.48 | 0.32 | 0.34 | -0.09 | 0.35 | 0.35 | 0.12 | 0.21 | 0.21 | 0.17 | 0.36 |
|  | PPV (%) | 52.3 | 84.2 | 70.4 | 25.5 | 63.6 | 81.8 | 85.7 | 100.0 | 66.7 | 75.0 | 52.0 |
|  | NPV (%) | 87.9 | 79.7 | 80.7 | 45.5 | 81.3 | 80.7 | 75.2 | 77.2 | 77.3 | 76.3 | 82.8 |
|  | ACC (%) | 74.4 | 80.2 | 79.1 | 26.7 | 77.9 | 80.8 | 75.6 | 78.5 | 76.2 | 76.2 | 73.8 |
| ≥60 Y | Sn (%) | 42.6 | 17.0 | 23.4 | 91.5 | 24.5 | 22.3 | 5.3 | 8.5 | 11.7 | 6.4 | 35.1 |
| n=256 | Sp (%) | 76.5 | 96.9 | 93.8 | 2.5 | 85.2 | 93.8 | 97.5 | 98.8 | 96.9 | 100.0 | 82.7 |
|  | Y's I | 0.19 | 0.14 | 0.17 | -0.06 | 0.10 | 0.16 | 0.03 | 0.07 | 0.09 | 0.06 | 0.18 |
|  | PPV (%) | 51.3 | 76.2 | 68.8 | 35.2 | 48.9 | 67.7 | 55.6 | 80.0 | 68.8 | 100.0 | 54.1 |
|  | NPV (%) | 69.7 | 66.8 | 67.9 | 33.3 | 66.0 | 67.6 | 64.0 | 65.0 | 65.4 | 64.8 | 68.7 |
|  | ACC (%) | 64.1 | 67.6 | 68.0 | 35.2 | 62.9 | 67.6 | 63.7 | 65.6 | 65.6 | 65.6 | 65.2 |
| Female | | | | | | | | | | | | |
| ＜60 Y | Sn (%) | 24.2 | 9.1 | 7.6 | 78.8 | 7.6 | 10.6 | 4.5 | 3.0 | 6.1 | 7.6 | 16.7 |
| n=207 | Sp (%) | 92.9 | 97.9 | 99.3 | 15.6 | 95.7 | 98.6 | 99.3 | 99.3 | 98.6 | 99.3 | 95.0 |
|  | Y's I | 0.17 | 0.07 | 0.07 | -0.06 | 0.03 | 0.09 | 0.04 | 0.02 | 0.05 | 0.07 | 0.12 |
|  | PPV (%) | 61.5 | 66.7 | 83.3 | 30.4 | 45.5 | 77.8 | 75.0 | 66.7 | 66.7 | 83.3 | 61.1 |
|  | NPV (%) | 72.4 | 69.7 | 69.7 | 61.1 | 68.9 | 70.2 | 69.0 | 68.6 | 69.2 | 69.7 | 70.9 |
|  | ACC (%) | 71.0 | 69.6 | 70.0 | 35.7 | 67.6 | 70.5 | 69.1 | 68.6 | 69.1 | 70.0 | 70.0 |
| ≥60 Y | Sn (%) | 29.5 | 15.2 | 15.2 | 89.5 | 20.0 | 13.3 | 7.6 | 9.5 | 8.6 | 16.2 | 25.7 |
| n=193 | Sp (%) | 85.2 | 95.5 | 90.9 | 8.0 | 94.3 | 95.5 | 98.9 | 100.0 | 96.6 | 96.6 | 90.9 |
|  | Y's I | 0.15 | 0.11 | 0.06 | -0.03 | 0.14 | 0.09 | 0.06 | 0.10 | 0.05 | 0.13 | 0.17 |
|  | PPV (%) | 70.5 | 80.0 | 66.7 | 53.7 | 80.8 | 77.8 | 88.9 | 100.0 | 75.0 | 85.0 | 77.1 |
|  | NPV (%) | 50.3 | 48.6 | 47.3 | 38.9 | 49.7 | 48.0 | 47.3 | 48.1 | 47.0 | 49.1 | 50.6 |
|  | ACC (%) | 54.9 | 51.8 | 49.7 | 52.3 | 53.9 | 50.8 | 49.2 | 50.8 | 48.7 | 52.8 | 55.4 |
| Data are shown as percentages or absolute numbers. ACC=diagnostic accuracy; NPV=negative predictive value; PPV=positive predictive value; Sn=Sensitivity; Sp=Specificity; Y's I=Youden's Index. | | | | | | | | | | | | |
|
