# Supplementary material for: Models for improved diagnosis of left ventricular hypertrophy based on conventional electrocardiographic criteria
Source: BMC Cardiovasc Disord. 2017 Aug 8;17:217. doi: 10.1186/s12872-017-0637-8 (PMC5549337; doi:10.1186/s12872-017-0637-8)
Supplement: Supplementary file 5 — AUC of the 22 conventional ECG criteria for gender, age, and BMI. The AUC of the 22 conventional ECG criteria in male and female groups, <60 years old and ≥60 years old groups, and BMI <25 kg/m2 and BMI ≥ 25 kg/m2 groups; <60 years old male and ≥60 years old male groups, and <60 years old female and ≥60 years old female groups. (DOC 55 kb) [file 12872_2017_637_MOESM5_ESM.doc]

**Additional file 5: Table S5 AUC of the 22 conventional ECG criteria for gender, age, and BMI.**

| ECG criteria | Male | Female | ＜60 Y | ≥60 Y | BMI＜25 kg/m^2^ | BMI≥25kg/m^2^ | ＜60 Y male | ≥60 Y male | ﹤60 Y female | ≥60 Y female |
| --- | --- | --- | --- | --- | --- | --- | --- | --- | --- | --- |
| ECG 1 | 0.55 | 0.57 | 0.565 | 0.549 | 0.572 | 0.537 | 0.553 | 0.544 | 0.578 | 0.533 |
| ECG 2 | 0.575 | 0.605 | 0.586 | 0.584 | 0.622 | 0.533 | 0.591 | 0.564 | 0.579 | 0.585 |
| ECG 3 | 0.594 | 0.621 | 0.605 | 0.602 | 0.651 | 0.532 | 0.628 | 0.574 | 0.587 | 0.612 |
| ECG 4 | 0.551 | 0.602 | 0.574 | 0.562 | 0.594 | 0.537 | 0.539 | 0.55 | 0.601 | 0.557 |
| ECG 5 | 0.495 | 0.486 | 0.494 | 0.507 | 0.496 | 0.491 | 0.563 | 0.471 | 0.44 | 0.553 |
| ECG 6 | 0.559 | 0.579 | 0.58 | 0.578 | 0.602 | 0.521 | 0.645 | 0.528 | 0.532 | 0.608 |
| ECG 7 | 0.52 | 0.554 | 0.549 | 0.541 | 0.553 | 0.51 | 0.604 | 0.487 | 0.516 | 0.591 |
| ECG 8 | 0.598 | 0.565 | 0.614 | 0.568 | 0.559 | 0.613 | 0.64 | 0.592 | 0.608 | 0.533 |
| ECG 9 | 0.616 | 0.627 | 0.622 | 0.603 | 0.611 | 0.608 | 0.633 | 0.629 | 0.653 | 0.598 |
| ECG 10 | 0.697 | 0.627 | 0.673 | 0.621 | 0.66 | 0.641 | 0.787 | 0.646 | 0.618 | 0.601 |
| ECG 11 | 0.718 | 0.674 | 0.688 | 0.651 | 0.695 | 0.64 | 0.8 | 0.685 | 0.671 | 0.649 |
| ECG 12 | 0.717 | 0.666 | 0.688 | 0.646 | 0.692 | 0.637 | 0.803 | 0.681 | 0.662 | 0.638 |
| ECG 13 | 0.692 | 0.653 | 0.678 | 0.641 | 0.674 | 0.64 | 0.77 | 0.665 | 0.648 | 0.628 |
| ECG 14 | 0.696 | 0.667 | 0.644 | 0.62 | 0.669 | 0.609 | 0.751 | 0.666 | 0.639 | 0.627 |
| ECG 15 | 0.519 | 0.51 | 0.492 | 0.479 | 0.505 | 0.495 | 0.498 | 0.524 | 0.502 | 0.476 |
| ECG 16 | 0.674 | 0.63 | 0.644 | 0.606 | 0.665 | 0.597 | 0.763 | 0.616 | 0.592 | 0.623 |
| ECG 17 | 0.695 | 0.665 | 0.669 | 0.629 | 0.674 | 0.626 | 0.77 | 0.666 | 0.665 | 0.629 |
| ECG 18 | 0.692 | 0.634 | 0.655 | 0.625 | 0.681 | 0.602 | 0.781 | 0.632 | 0.587 | 0.641 |
| ECG 19 | 0.681 | 0.616 | 0.655 | 0.623 | 0.675 | 0.589 | 0.783 | 0.619 | 0.569 | 0.628 |
| ECG 20 | 0.642 | 0.63 | 0.619 | 0.614 | 0.639 | 0.592 | 0.707 | 0.621 | 0.589 | 0.639 |
| ECG 21 | 0.691 | 0.657 | 0.654 | 0.637 | 0.656 | 0.639 | 0.691 | 0.696 | 0.67 | 0.59 |
| ECG 22 | 0.681 | 0.649 | 0.633 | 0.634 | 0.665 | 0.609 | 0.744 | 0.647 | 0.598 | 0.651 |
